# ETDRS grading with CLARUS ultra-widefield images shows agreement with 7-fields colour fundus photography

**DOI:** 10.1186/s12886-024-03537-z

**Published:** 2024-09-03

**Authors:** Ana Rita Santos, Sejal Ghate, Marta Lopes, Ana Cláudia Rocha, Torcato Santos, Débora Reste-Ferreira, Niranchana Manivannan, Katharina Foote, José Cunha-Vaz

**Affiliations:** 1 AIBILI - Association for Innovation and Biomedical Research on Light and Image, Coimbra, Portugal; 2 CORC – Coimbra Ophthalmology Reading Centre, Coimbra, Portugal; 3 Center for Translational Health and Medical Biotechnology Research (TBIO)/Health Research Network (RISE-Health), ESS, Polytechnic of Porto, Porto, Portugal; 4 Carl Zeiss Meditec, Inc, Dublin, CA USA; 5 Faculty of Medicine, University of Coimbra, Coimbra, Portugal

**Keywords:** Diabetic retinopathy, Colour fundus photography, Ultra-widefield imaging, CLARUS, ETDRS severity scale

## Abstract

**Background:**

To analyse and compare the grading of diabetic retinopathy (DR) severity level using standard 35° ETDRS 7-fields photography and CLARUS™ 500 ultra-widefield imaging system.

**Methods:**

A cross-sectional analysis of retinal images of patients with type 2 diabetes (*n* = 160 eyes) was performed for this study. All patients underwent 7-fields colour fundus photography (CFP) at 35° on a standard Topcon TRC-50DX^®^ camera, and ultra-widefield (UWF) imaging at 200° on a CLARUS™ 500 (ZEISS, Dublin, CA, USA) by an automatic montage of two 133° images (nasal and temporal). 35° 7-fields photographs were graded by two graders, according to the Early Treatment Diabetic Retinopathy Study (ETDRS). For CLARUS UWF images, a prototype 7-fields grid was applied using the CLARUS review software, and the same ETDRS grading procedures were performed inside that area only. Grading of DR severity level was compared between these two methods to evaluate the agreement between both imaging techniques.

**Results:**

Images of 160 eyes from 83 diabetic patients were considered for analysis. According to the 35° ETDRS 7-fields images, 22 eyes were evaluated as DR severity level 10–20, 64 eyes were evaluated as DR level 35, 41 eyes level 43, 21 eyes level 47, 7 eyes level 53, and 5 eyes level 61. The same DR severity level was achieved with CLARUS 500 UWF images in 92 eyes (57%), showing a perfect agreement (k > 0.80) with the 7-fields 35° technique. Fifty-seven eyes (36%) showed a higher DR level with CLARUS UWF images, mostly due to a better visualization of haemorrhages and a higher detection rate of intraretinal microvascular abnormalities (IRMA). Only 11 eyes (7%) showed a lower severity level with the CLARUS UWF system, due to the presence of artifacts or media opacities that precluded the correct evaluation of DR lesions.

**Conclusions:**

UWF CLARUS 500 device showed nearly perfect agreement with standard 35° 7-fields images in all ETDRS severity levels. Moreover, CLARUS images showed an increased ability to detect haemorrhages and IRMA helping with finer evaluation of lesions, thus demonstrating that a UWF photograph can be used to grade ETDRS severity level with a better visualization than the standard 7-fields images.

**Trial registration:**

Approved by the AIBILI - Association for Innovation and Biomedical Research on Light and Image Ethics Committee for Health with number CEC/009/17- EYEMARKER.

## Introduction

Diabetic Retinopathy (DR) is a complication of diabetes that can lead to vision loss. This disease has a great impact in the working age population (20–74 years) and it is still the main cause of blindness across the world [1]. Several imaging tools were developed in the past years to improve the diagnosis and follow-up of the disease, but seven-fields colour fundus photography (CFP) is still considered the gold standard method to assess DR severity using the Early Treatment Diabetic Retinopathy Study (ETDRS) disease severity scale, which is the most commonly used classification for clinical trials [2, 3].

The ETDRS DR severity scale is based on the number of lesions that appear in CFP obtained in seven different locations of the retina [4]. However, the acquisition of 7-fields photography frequently requires extensive and prolonged photographer training and certification by external reading centres to be accepted for clinical trials. The procedure itself requires sufficient dilation of the eye pupil and cooperation of patients to follow a fixation point. It can be challenging to obtain well-focused images in peripheral gaze positions, especially if there are media opacities present.

Additionally, the grading process of the ETDRS 7-fields images can be extremely labour-intensive, depending on the quality of the pictures, presence of artifacts, focus, and sharpness of the peripheral fields. Well-trained graders are a necessity to identify and recognize features related to DR that can be very subtle or easily unnoticed in the reddish background of the retina.

Seven-fields photography with the standard ETDRS protocol are generally acquired with 30°- 40° of field of view (FOV), reaching about nearly 35% of the whole retinal area [5–7]. However, the retinal periphery may also be affected in diabetic patients [5, 8], which is overlooked by this technique. The more recent development of ultra-widefield (UWF) imaging, as provided by devices such as CLARUS™ 500 (ZEISS, Dublin, CA, USA) or Optos™ (Optos plc., Dunfermline, UK), have allowed not only a greater visualization of the peripheral retina in just one or two images (more than 80%) [5, 9], but also decrease fatigue and discomfort of patients. These recent imaging tools also overcome the quality and retinal area delimitation inconsistencies described above, key factors to improve the ability to visualize features related to DR pathology [9].

With the introduction of UWF imaging, some manufacturers began to specify their systems’ FOV capabilities using the angle subtended at the centre of the spherical eye instead of the traditional visual angle used in conventional fundus cameras. Visual angle is defined as the angle subtended at the exit of pupil of the eye [10]. Taking into consideration the eye dimensions, as well as the refractive difference between air and the eye mediums, a conversion factor of ~ 1.5 was derived to convert eye to visual angles. However, as shown by Yao et al. [11], this is only reasonable for angle range near the eye axis. A more detailed analysis showed that this conversion factor is non-linear and can change from 1.51 in narrow FOV systems to 1.34 in UWF systems. For simplicity, the FOV of conventional fundus cameras will be referred using the standard visual angle while UWF system FOV as the angle subtended at the centre of the spherical eye, as manufacturers do.

UWF equipment like Optos can acquire approximately 80% of the retina in one image without the need for pupil dilation by using ultra-widefield scanning laser ophthalmoscopy (SLO) technology [9]. However, the final image is based on the superimposition of two images acquired with 2 different laser wavelengths: a green and a red wavelength, yielding a colour image that despite its high contrast and sharpness, gives the retina a greenish and unrealistic aspect. Also, this 200° image is usually disturbed by artifacts caused by the presence of eyelashes or eyelids, obscuring the retina peripheral area, and causing misinterpretation of lesions that may lead to inaccurate DR severity evaluation.

CLARUS 500 can capture 133° of retinal FOV in a single image [11], achieving 200° ultra-widefield with the acquisition of just two images (a temporal and nasal image of the retina) [12]. This device uses a technology called Broad Line Fundus Imaging (BLFI) which makes use of scanned red, green, and blue light-emitting diodes that sequentially illuminate the retina to generate true colour and reduced haze allowing a larger, clear view of the retina than typical traditional fundus cameras. The design also allows a single exposure to image an area of the retina previously covered by the 7-fields in the ETDRS definition.

Several studies have suggested moderate to substantial agreement between Optos UWF and ETDRS 7-fields imaging [13, 14]. However, there is only limited data regarding the validity of DR assessment using the CLARUS 500 instrument using a small patient dataset [15, 16].

The purpose of the present study is to evaluate the agreement between CLARUS 500 UWF photography and the standard 35° 7-field CFP in the assessment of ETDRS DR severity level.

## Methods

### Subjects

This is an observational, cross-sectional, and single centre study in type 2 diabetes (T2D) patients to evaluate the agreement of DR severity level among ETDRS 7-fields fundus images at 35° and UWF images at 200°.

Patients were recruited from the AIBILI (Association for Innovation and Biomedical Research on Light and Image) electronic medical record under the scope of the ongoing project EYEMARKER - “Characterization of potential biomarkers of Eye Disease and Vision Loss” (CEC/009/17). This study was approved by the local ethics committee for health and the tenets of the Declaration of Helsinki were followed. Written informed consent to participate in the study was provided by each participant, after all procedures were explained. All subjects underwent a complete ophthalmic examination including best-corrected visual acuity (BCVA), intraocular pressure, slit-lamp bio-microscopy, fundus imaging - CFP and UWF fundus photography. The following exclusion criteria were applied: (1) media opacities or other ocular conditions that preclude or may interfere with fundus examinations (i.e.: mature cataract, corneal or iris diseases, vitreous haemorrhage, etc.) ; (2) age-related macular degeneration, advanced glaucoma, vitreomacular disease, or other retinal vascular disease; (3) any ocular condition that, in the opinion of the investigator, may affect retinopathy status or visual acuity.

### Demographic and ophthalmological data

Demographics including age, duration of diabetes, comorbidities, and concomitant medication were collected for each participant. Physical assessment with biometric measures (body weight and height) and blood pressure evaluation were also collected. Laboratory analyses included plasma concentrations of haemoglobin (HbA1c), lipid fractionation identifying total cholesterol, high-density lipoprotein (HDL), low-density lipoprotein (LDL), and triglycerides were measured to assess patient’s metabolic control.

#### 35°ETDRS 7-fields colour fundus photography

Dilation of pupil was performed in all patients before fundus imaging with 0.5% tropicamide and 0.5% phenylephrine hydrochloride eye drops. 7-fields CFP images were obtained at 35° in dilated patients using a Topcon TRC-50DX^®^ mydriatic retinal camera (Topcon Medical Systems, Tokyo, Japan) with a resolution of 3596 × 2448 pixels and following the ETDRS report number 10 acquisition protocol [4].

#### CLARUS 500 photography

**Fig. 1:**
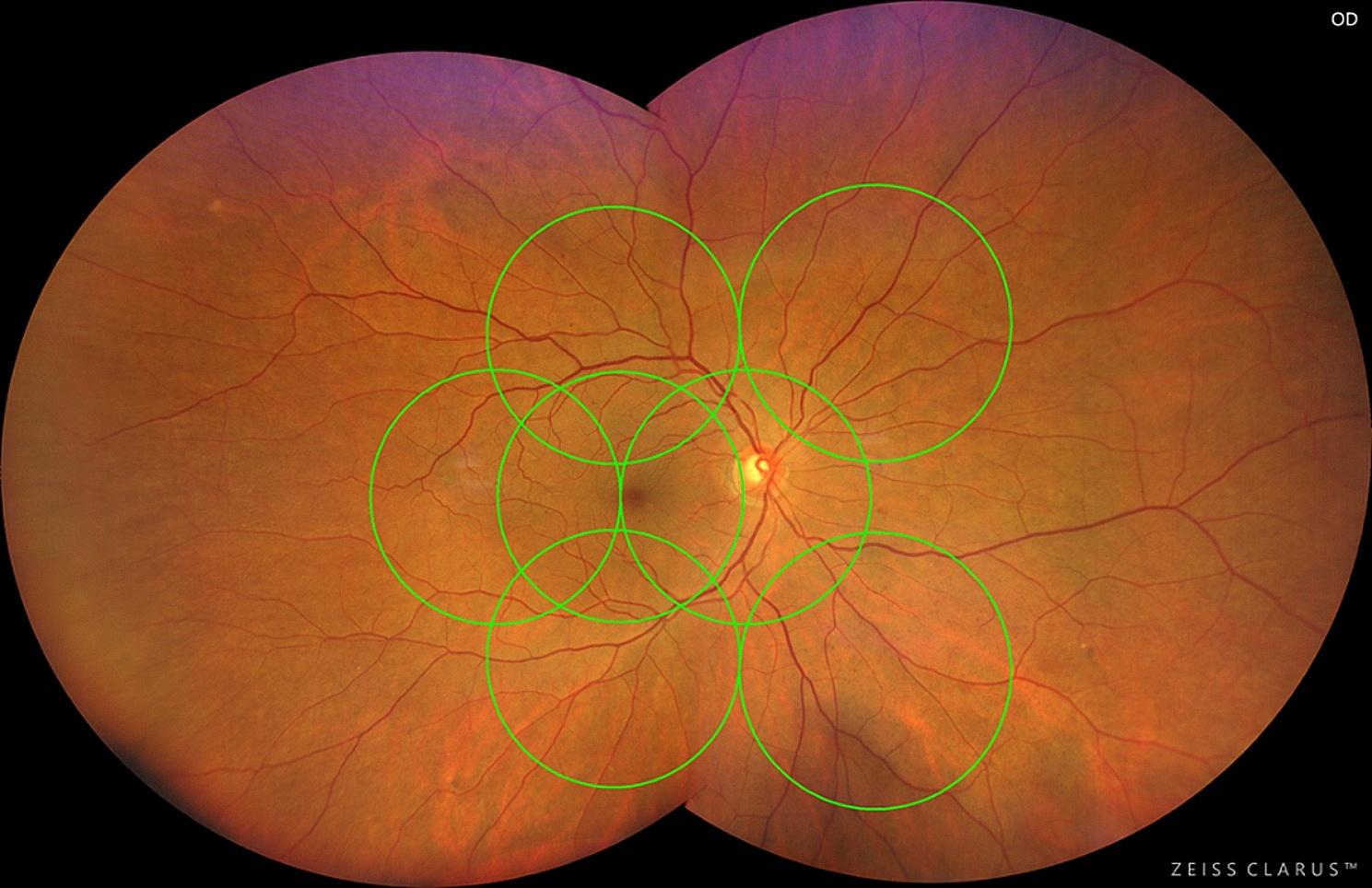
Representative image of a CLARUS 500 acquisition with the montage of the nasal and temporal images, with the ETDRS 7-fields grid superimposed

After the acquisition of standard ETDRS 7-fields photography images, patients underwent CLARUS 500 UWF imaging using the UWF acquisition option. This option allows the acquisition of 2 images with 133° FOV each, one temporal to the fovea and one nasal to the optic disc. After the acquisition, an automatic montage of both images into a composition of approximately 200° FOV was performed by the software, based on the position of the optic nerve head in both images (see Fig. 1).

### Imaging grading

ETDRS 7-fields images were exported to a centralized reading centre - Coimbra Ophthalmology Reading Centre (CORC) and classified by two independent graders using the ETDRS severity scale to compare the presence and severity of DR lesions with the ETDRS study standard photos [4].

CLARUS 500 2-image montages were exported from the device and imported into the CLARUS reviewer software (version: 1.2.0.56075). A grid with the equivalent standard 7-fields area (ETDRS grid) was superimposed on the 2-image montage using the optic nerve head as a reference. Independent graders reviewed the images and classified the area inside the ETDRS grid using the same ETDRS DR severity scale described above. The grading of CLARUS UWF images was made with a time interval of more than 7 days after the classification of the 35° ETDRS 7-fields images to avoid memory bias from previous classification. In the case of grading discrepancies between the two retinal specialists, these were arbitrated by another senior retinal specialist who resolved the issue by also grading the images in question.

### Statistical analysis

All data was analysed statistically using Stata 16.1 (StataCorp LLC, College Station, TX, USA). Demographic characteristics were summarized as mean values and corresponding standard deviations for continuous variables. Weighted kappa and Gwet’s AC statistics were performed to assess inter-rater agreement between the two independent specialists for both imaging modalities (35° ETDRS 7-fields and CLARUS) and also to evaluate the (agreement) DR severity grading between both imaging techniques (CLARUS versus 35° ETDRS 7-fields). The 95% confidence interval (CI) were also calculated for both measures. The Landis and Koch guidelines were adopted for interpreting kappa statistics: ≤ 0.2 slight agreement; 0.21 to 0.40, fair agreement; 0.41 to 0.60, moderate agreement; 0.61 to 0.80, substantial agreement; and ≥ 0.81 almost perfect agreement [17].

## Results

A total of 160 eyes from 83 patients with type 2 diabetes were considered for analysis. The mean age of patients was 67.19 ± 9.32 years with 80% being male. Patients’ demographic characteristics are summarized in Table 1 along with their distribution by ETDRS severity levels.

According to the 35° ETDRS 7-fields images, 15 eyes were considered as no DR (ETDRS level 10–15), 7 eyes were considered having only microaneurysms (ETDRS level 20), 64 eyes were considered having mild Non-Proliferative DR (NPDR) (ETDRS level 35), 41 eyes presented moderate NPDR (ETDRS level 43), 21 eyes moderately severe NPDR (ETDRS level 47), 7 eyes severe NPDR (ETDRS level 53) and 5 eyes Proliferative DR (ETDRS level 61 or 65).

**Table 1:**
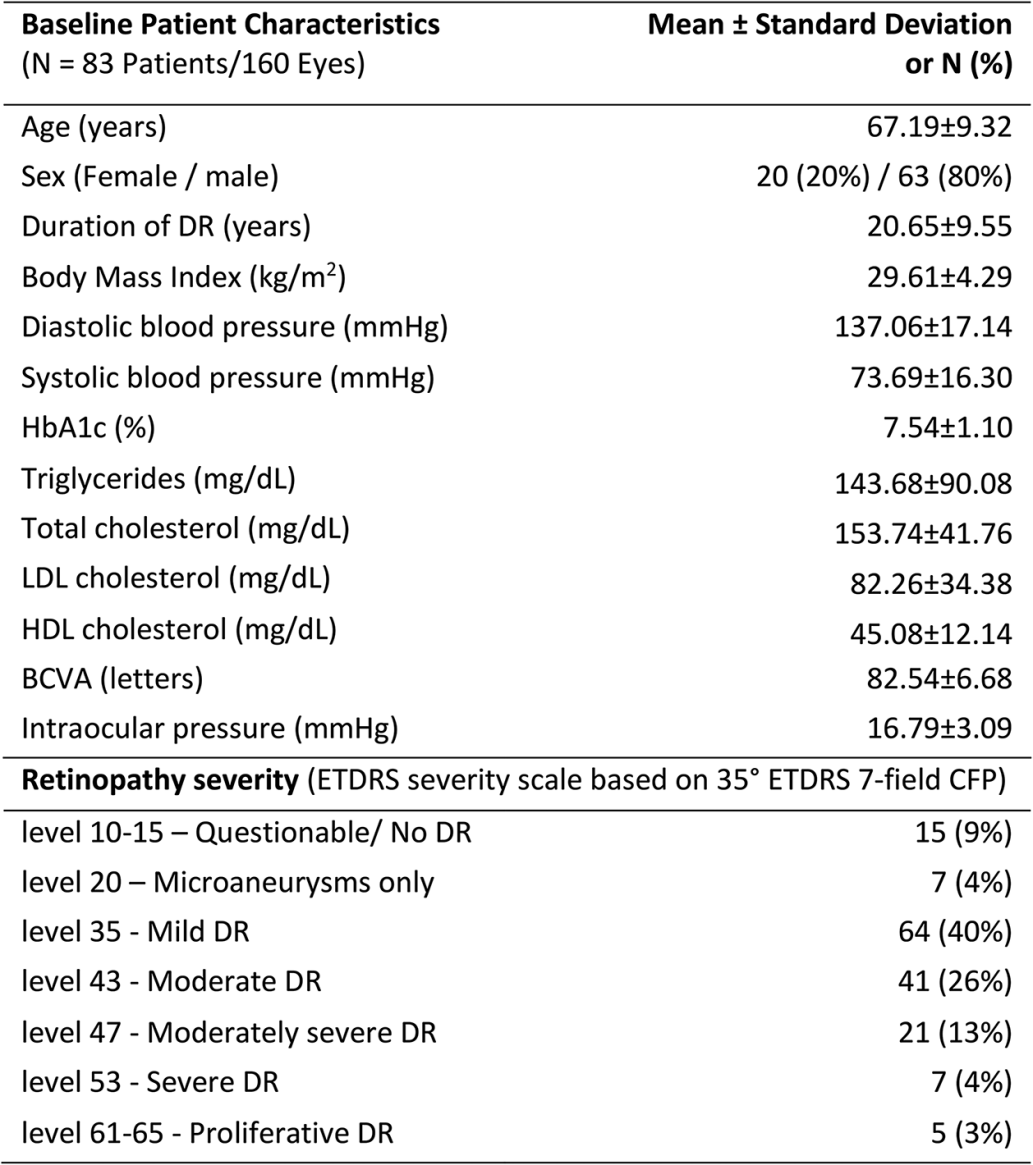
Study patients characteristics and DR severity distributions

### Agreement between two independent graders in 35° ETDRS 7-fields and CLARUS 500 images

Our results showed that the agreement of DR severity between the two independent graders was almost perfect in both 7-fields CFP and CLARUS images (k > 0.80). For 35° ETDRS 7-fields images, the weighted Kappa was 0.858 [95% confidence interval (CI) CI, 0.793–0.922] whereas for Gwet’s AC was 0.928 [95% CI, 0.894–0.961]. Similar results between the two graders were also showed in CLARUS images, the weighted Kappa was 0.898 [95% confidence interval CI, 0.856–0.940] whereas for the Gwet’s AC was 0.938 [95% CI, 0.914–0.963].

### Agreement in ETDRS DR severity between 35° ETDRS 7-fields and CLARUS 500

**Fig. 2:**
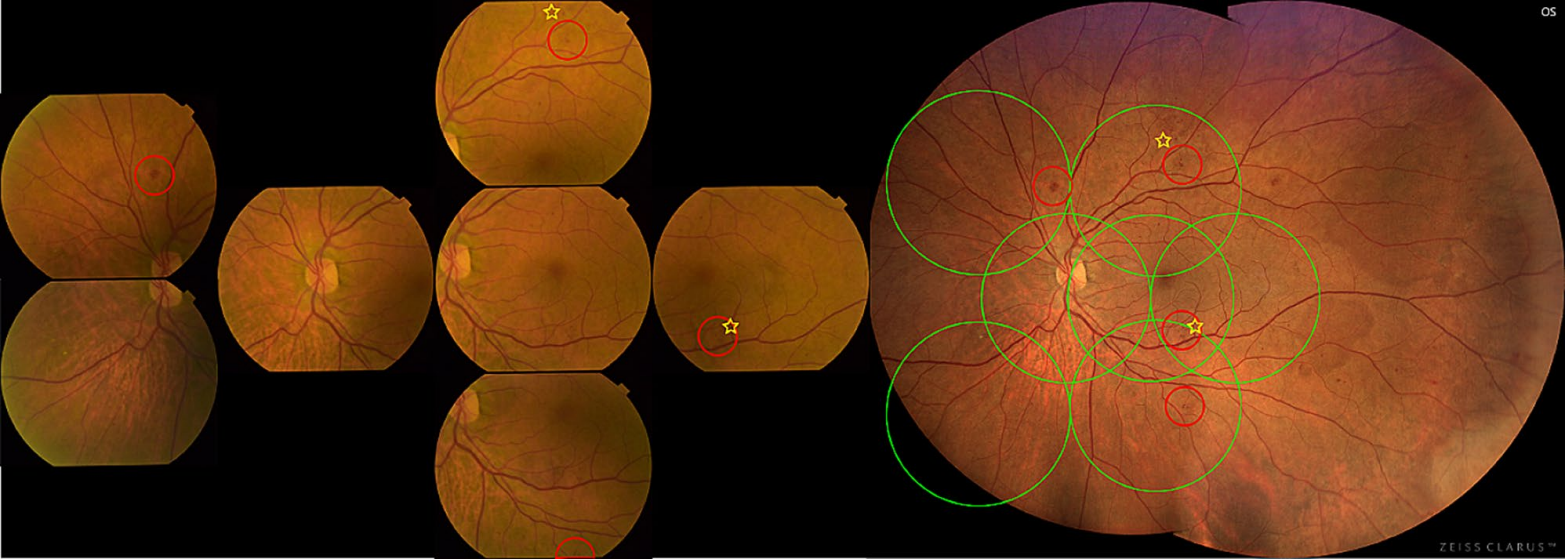
35° ETDRS 7-fields images and CLARUS 500 images of a patient graded with the same DR level in both devices − 47 A (moderate Haemorrhages and Microaneurysms (red circles) in 4 fields and IRMA definite present in 2 fields (yellow star)

A comparison of the DR severity levels between 35° ETDRS 7-fields images *versus* CLARUS 500 UWF images was also assessed. The agreement between both imaging modalities was almost perfect (Gwet’s AC 0.8128 [95% CI, 0.773–0.852]). Our results showed that 57% of all cases (92 of 160 eyes) were classified with the same DR severity level in both imaging techniques (Table 2A. and Fig. 2).

**Table 2A:**
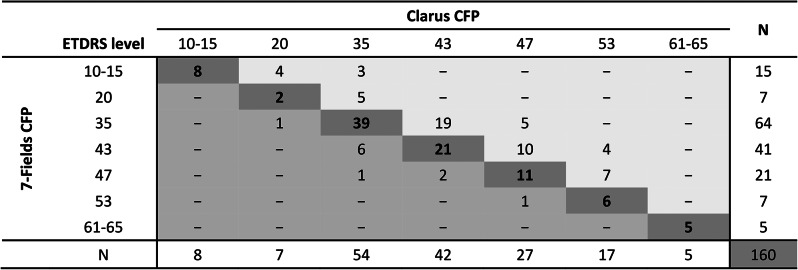
Distribution of subjects showing lower, higher, or same ETDRS grading in CLARUS 500 compared to 35°ETDRS 7-Fields CFP

**Footnote**: The bold values represent the eyes with same severity levels in 35° ETDRS 7-fields CFP and CLARUS, corresponding to 57% of the cases (92/160 eyes)

**Table 2B:**
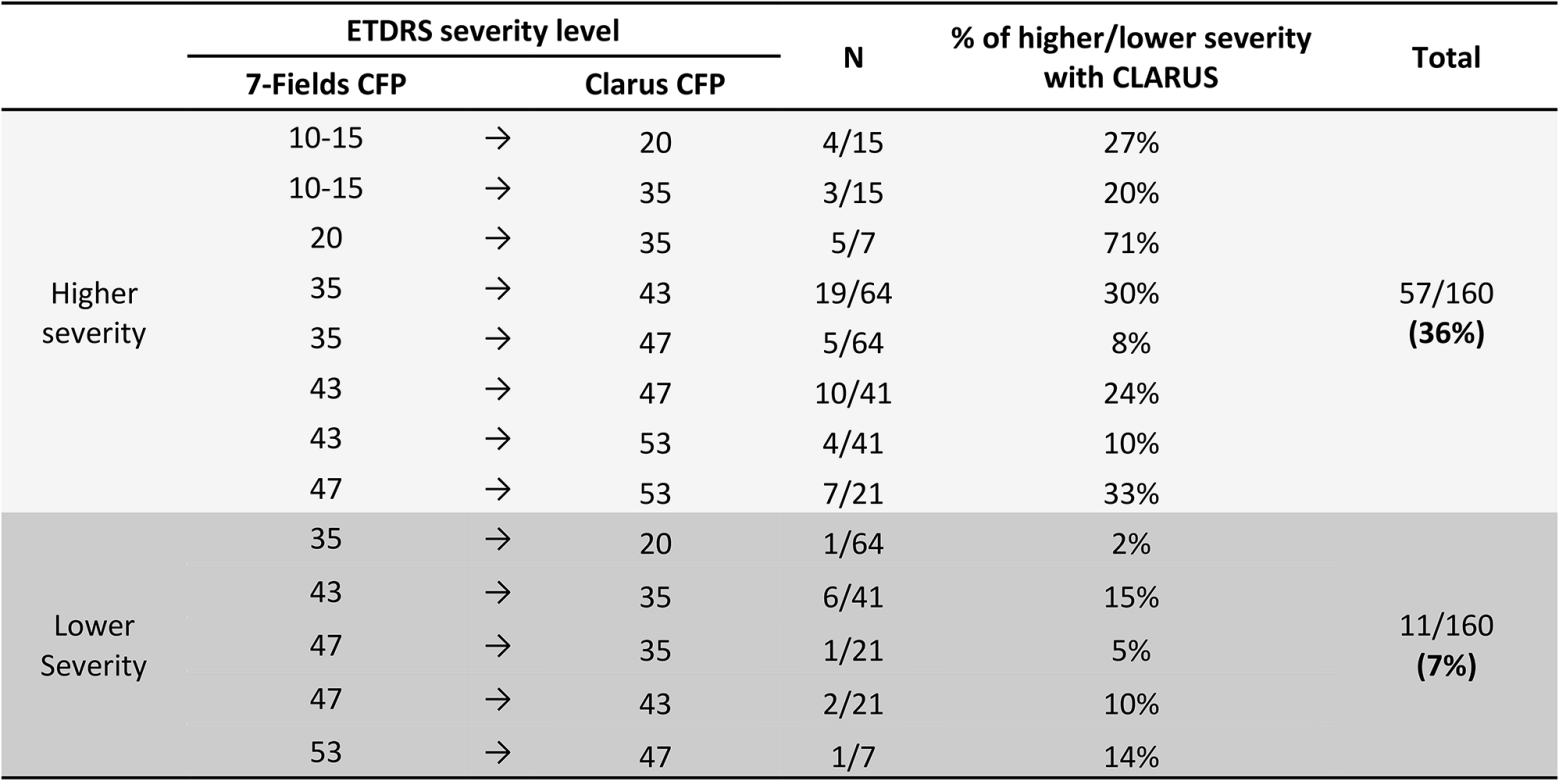
The percentages associated with disagreement of ETDRS grading between techniques

**Footnote**: 36% (57/160 eyes) of the cases showed a higher severity level, whereas only 7% (11/160 eyes) of the cases showed a lower level when comparing 35° ETDRS 7-fields with CLARUS.

### Discrepancies in DR Severity between 35° ETDRS 7-fields and CLARUS 500

**Fig. 3:**
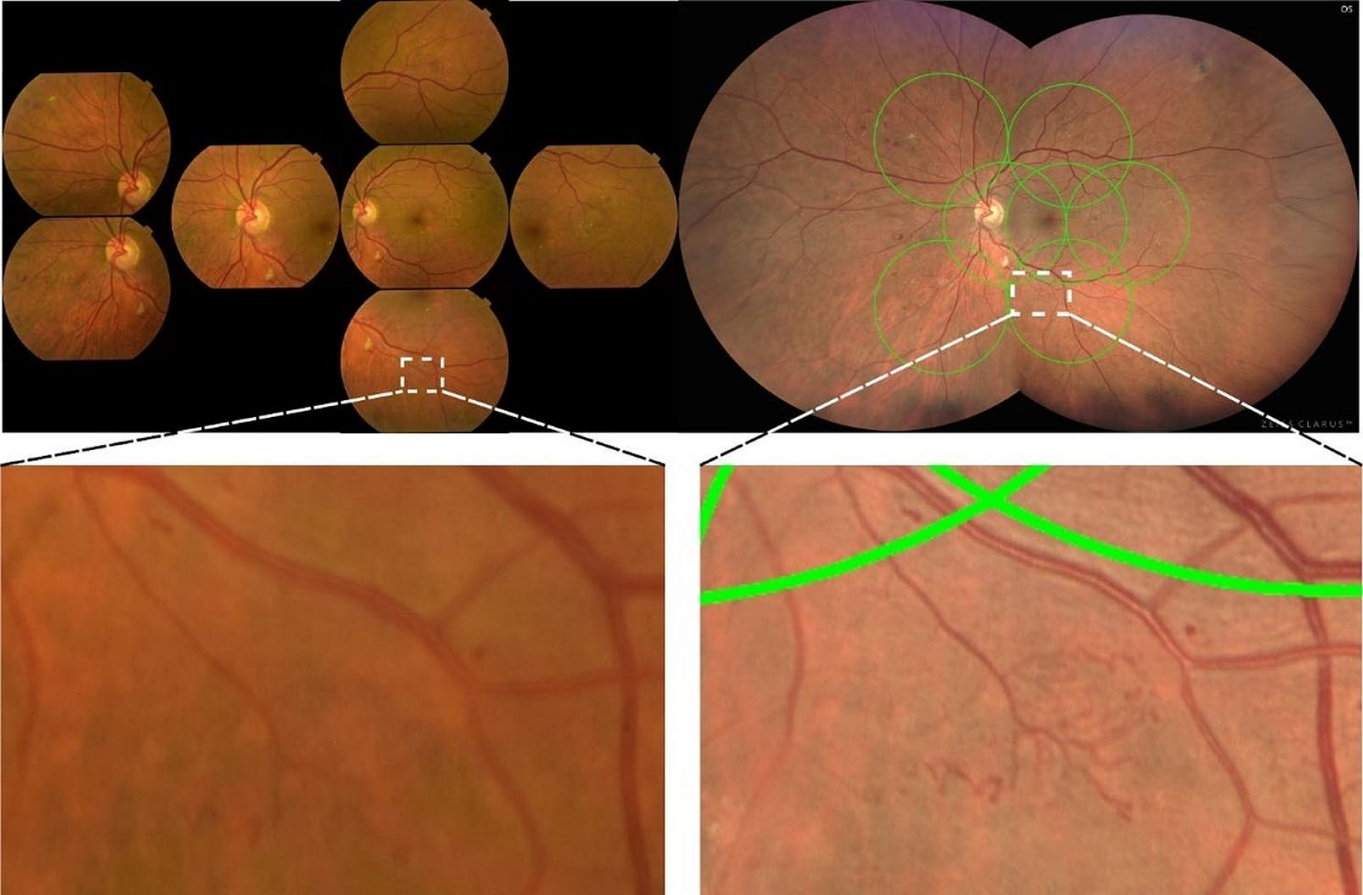
On the top row 35° ETDRS 7-fields images of a patient graded as DR level 47 A (with moderate Haemorrhages and Microaneurysms in 4 fields and IRMA definite present in 2 fields) and graded as 53 C in CLARUS 500 images (moderate IRMA in one field). Bottom row, magnified areas highlighting the distinct severity of the IRMA lesion

CLARUS 500 UWF images showed a higher DR severity level in 57 eyes (36%). The reasons for this increase of severity in the UWF images were mostly related to a higher detection rate of haemorrhages (12/57 eyes – 21%), intraretinal microvascular abnormalities (IRMA) (19/57 eyes – 33%) and improved visualization and characterization of these same lesions causing an increase of their severity in 45 eyes (79%). A change of DR severity from level 35 to level 43 or 47 was registered in 38% of the cases (24/64 eyes), from DR severity level 43 to level 47 or 53 in 34% of the cases (14/41 eyes) and from DR severity level 47 to level 53 in 33% eyes (7/21 eyes) (Table 2B and Fig. 3).

Only 7% (*n* = 11/160) of all cases showed a decrease in severity level with CLARUS 500 images (Table 2B). This was mainly due to features being concealed by montage artifacts and presence of cortical cataracts obscuring the most peripheral areas of the image.

## Discussion

The agreement between two imaging methods for DR detection and staging was evaluated in this study: the 35° EDTRS 7-fields photography and the CLARUS 500 UWF images at 200°. An almost perfect agreement on DR severity was found between these two imaging methods in all DR severity stages.

These results show that UWF imaging devices can be a useful alternative to the laborious and unfriendly method of acquiring 7 different images of the peripheral retina with only 30° FOV and documenting only 35% of the entire retina surface.

Indeed, despite the ETDRS 7-fields imaging technique being still the gold standard to evaluate DR severity level, it is difficult to use in clinical practice and is mainly used in clinical trials mainly to ensure the standardization of procedures and inclusion criteria, along with the comparability of results among different countries and populations. Even so, difficulties in mastering this photographic technique are observed in different investigational sites and reading centres that frequently report important quality issues in the images and constraints in the training and certification of technicians [18]. 30° ETDRS 7-fields photography is strongly dependent on a patient’s dilation and collaboration, but also on the photographer’s skill to obtain focused images with correct field definition and absence of artifacts. 30°retinal images are highly affected by media opacities or corneal defects as their small FOV limits possible adjustments to take a perfectly focused image around these features.

The emergence of widefield devices also increased its preference by sites for this kind of fundus photography since in just one shot, technicians can easily capture a good image and physicians can evaluate the entire retina (centre and periphery) at a single glance. In most cases, dilation is not required, and patients just need to look in one direction to get the entire retina documented. It expedites the daily practice workflow with a superior quality and improved visualization of central but also peripheral lesions [19].

Furthermore, our study demonstrated that CLARUS 500 images detected a higher ETDRS level in 36% of the eyes. This increase in severity was mainly due to enhanced visualization of haemorrhages, which improved their detection and highlighted higher severity. Additionally, the improved ability to detect other DR lesions such as IRMA, abnormal branching, or dilation of existing blood capillaries, which can be very thin and difficult to differentiate against the reddish background of the retina, contributed to this finding. These results indicate that CLARUS 500 images offer a higher resolution, better clarity, definition, fundus contrast, and better visualization than 30° ETDRS 7-fields in the detection and characterization of DR lesions. This is primarily because CLARUS devices feature true colour imaging combining red, green, and blue scanning light sources in BLFI technology with a high-resolution of 7.3 microns. Additionally, fundus images obtained with this device are not influenced by mild cataracts, and its partially confocal optics can reduce artifacts in retinal images caused by eyelashes and eyelids [16].

These results are especially important for screening or telemedicine programs [20]. DR screening guidelines recommend referring patients for a more comprehensive ophthalmic examination in the presence of Moderate NPDR (presence of haemorrhages, microaneurysms, and IRMA in up to 3 quadrants – ETDRS level 43) and an urgent referral in the presence of severe NPDR (presence of haemorrhages in all four quadrants, venous beading in two or more quadrants, or IRMA in one or more quadrants – ETDRS levels ≥ 47) due to the risk of progression to proliferative DR [21, 22]. Our results showed that 24 of 160 cases (15%), changed from ETDRS level 35 to levels 43 or 47 and would be referred when imaged with the CLARUS 500 wide-field device, thereby improving their diagnosis and the likelihood of preventing disease progression. Despite several studies demonstrating a good agreement between the standard 30° ETDRS 7-fields CFP technique and the UWF devices in the evaluation of DR severity [16, 23, 24], the industry is still reluctant to accept this new technology in their multicentric clinical trials. One of the reasons is the ability of this technology to show 70% more periphery than the classic ETDRS 7-fields method and the possible impact of detecting predominantly peripheral lesions (PPL). Silva et al. and Xiao et al. [8, 24] suggested a more severe assessment of DR in 10-12% of eyes when adding the periphery analysis to the 7-fields area [25].

However, the use of built-in overlays already available in some UWF devices -software, as used in the present study to isolate the standard 7-fields area from the periphery, allows an independent analysis of the central area. While in this study the image overlay used only indicated the field boundaries, other overlays are opaque and conceal the image area outside the 7-fields area, making graders’ assessment only dependent of the visible area, guaranteeing that all patients are evaluated using the same criteria, regardless of the imaging method that was used.

Despite not being the focus of the present study, PPL should be considered as part of the analysis, as proposed by different authors that show that presence and extent of PPLs are associated with increased risk of DR progression over 4 years [8]. With just one or two captures, photographers can document approximately 80% of the retina with superior quality and resolution, free of artifacts and with a much lower training time than the standard 30° ETDRS 7-fields method. From the perspective of the reading centres, certification of photographers will be easier and faster as the quality issues are significantly reduced, and grading time will severely decrease, as grading of DR lesions in just one or two images is much simplified, without missing data caused by misplaced fields. Therefore, the use of UWF imaging devices in clinical practice, and also in clinical trials, should be disseminated and implemented as the new standard of care in the near future. The price of these UWF devices can be considered an obstacle for the wide-spread use of this technique, especially in small clinical sites. However, the existence of multimodal models with the ability of having more than one imaging modality (colour imaging combined with fundus autofluorescence or fluorescein angiography or even optical coherence tomography) can be advantageous to the institution for other clinical studies in the longer term, and a way of making profit of the investment.

The use of just one UWF device to evaluate the agreement of DR severity with 35° ETDRS 7-fields images is a limitation of the present study, as well as the non-analysis of the peripheral retina, outside the ETDRS 7-fields area, to understand the impact of PPL in the disease staging. However, we were able to demonstrate that widefield 200° CLARUS images can be used to evaluate DR ETDRS severity level in an accurate and efficient manner. Another limitation of the current study is the relatively small number of patients as well as the imbalance between severity groups. Although all NPDR severity stages, as well as mild to moderate proliferative DR (severity levels 61 and 65) cases were covered, patients with high-risk proliferative DR (DR severity levels 71/75) were not included in this study since these were not available at our center.

## Conclusions

UWF CLARUS 500 device showed nearly perfect agreement with standard 35° 7-fields images in all ETDRS severity levels. CLARUS images showed an increased ability to detect haemorrhages and IRMA helping with finer evaluation of lesions demonstrating that a UWF photograph can be used to grade ETDRS severity level with a better visualization than the standard 7-fields images.

## Data Availability

All data generated or analyzed during this study are included in this article. Further inquiries can be directed to the corresponding author.

## Abbreviations

BCVA: Best-Corrected Visual Acuity
BLFI: Broad Line Fundus Imaging
CFP: Colour Fundus Photography
CI: Confidence Interval
DR: Diabetic Retinopathy
ETDRS: Early Treatment Diabetic Retinopathy Study
FOV: Field Of View
HbA1c: Haemoglobin
HDL: High-Density Lipoprotein
IRMA: Intraretinal Microvascular Abnormalities
LDL: Low-Density Lipoprotein
NPDR: Non-Proliferative DR
PPL: Predominantly Peripheral Lesions
SLO: Scanning Laser Ophthalmoscopy
T2D: Type 2 Diabetes
UWF: Ultra-Widefield
